# Assessment of Clinical Scales for Detection of Large Vessel Occlusion in Ischemic Stroke Patients from the Dijon Stroke Registry

**DOI:** 10.3390/jcm10245893

**Published:** 2021-12-15

**Authors:** Gauthier Duloquin, Mathilde Graber, Lucie Garnier, Sophie Mohr, Maurice Giroud, Catherine Vergely, Yannick Béjot

**Affiliations:** 1 Dijon Stroke Registry, EA7460, Pathophysiology and Epidemiology of Cerebro-Cardiovascular Diseases (PEC2), University of Burgundy, 21078 Dijon, France; gauthier.duloquin@chu-dijon.fr (G.D.); mathilde.graber@chu-dijon.fr (M.G.); lucie.garnier@chu-dijon.fr (L.G.); sophie.mohr@chu-dijon.fr (S.M.); maurice.giroud@chu-dijon.fr (M.G.); cvergely@u-bourgogne.fr (C.V.); 2 Department of Neurology, University Hospital of Dijon, 21000 Dijon, France

**Keywords:** ischemic stroke, registry, population studies, large vessel occlusion, scales

## Abstract

(1) Background: The limited availability of thrombectomy-capable stroke centres raises questions about pre-hospital triage of patients with suspected stroke (IS) due to large vessel occlusion (LVO). Aims: This study aimed to evaluate the diagnostic accuracy of clinical stroke severity scales available for LVO detection. (2) Methods: Patients with IS were prospectively identified among residents of Dijon, France, using a population-based registry (2013–2017). Clinical signs and arterial imaging data were collected. LVO was defined as an occlusion site affecting the terminal intracranial internal carotid artery, the M1 segment of the middle cerebral artery (MCA), or the basilar artery (restricted definition). A wide definition of LVO also included the M2 segment of the MCA. For each of the 16 evaluated scales, a receiver operator characteristic (ROC) analysis was performed, and the c-statistic representing the area under the ROC curve was evaluated to assess discrimination for predicting LVO. (3) Results: 971 patients were registered, including 123 patients (12.7%) with an LVO according to the restricted definition. The c-statistic for LVO detection ranged between 0.66 and 0.80 according to the different scales, with a sensibility varying from 70% to 98% and a specificity from 33% to 86%. According to the wide definition of LVO (174 patients, 17.9%), the c-statistic was slightly lower, ranging between 0.64 and 0.79. The sensitivity was 59% to 93%, and the specificity was 34% to 89%. (4) Conclusion: The clinical scales failed to combine a high sensitivity and a high specificity to detect LVO. Further studies are needed to determine the best strategy for pre-hospital triage of IS patients.

## 1. Introduction

Acute management of ischemic stroke (IS) has dramatically changed since the demonstration of the effectiveness of endovascular therapy with mechanical thrombectomy (MT) in patients with a large vessel occlusion (LVO) in the anterior circulation [1,2,3,4,5,6]. The current limited availability of thrombectomy-capable stroke centres, especially in rural areas, raises questions about the best care organization model to apply for the orientation of patients suspected of having a stroke. Two strategies have been proposed: the “drip and ship” model, referring to the transfer of patients to the closest hospital with either a primary stroke centre (i.e., a stroke centre that provides IV thrombolysis but not MT) or a telemedicine-equipped emergency department, with a subsequent transfer to a thrombectomy-capable stroke centre in case of LVO requiring MT; and the “mothership” model, consisting in a first-line transfer of patients to a thrombectomy-capable stroke centre. Although the “drip and ship” model can lead to avoidable delays in MT administration in patients with LVO, the “mothership” model is associated with an increased onset-to-needle time, as well as with a risk of addressing patients with no indication of MT in thrombectomy-capable stroke centres, thus leading to an excessive workload.

To attempt to address the issue of stroke patients’ triage, several clinical stroke severity scales have been developed, some of them with the aim of identifying patients with LVO. The reliability of these scales has been assessed in selected populations [7,8,9,10,11]. However, whether these scales have enough sensitivity and specificity remains to be explored in practice.

The aim of this study was to evaluate the diagnostic accuracy of these scales for LVO detection using data from a population-based stroke registry.

## 2. Materials and Methods

### 2.1. Study Population

Patients with an acute ischemic stroke (IS) that occurred between 1 January 2013 and 31 December 2017 were prospectively identified from the Dijon Stroke Registry [12,13,14]. This registry is an ongoing population-based study that complies with the criteria for conducting “ideal” incidence stroke studies [15] and the guidelines for reporting incidence and prevalence studies in neuroepidemiology according to the Standards of Reporting of Neurological Disorders (STROND) [16]. The methodology of the Dijon Stroke Registry has been described extensively elsewhere [12,13,14]. Its case collection relies on multiple overlapping sources of information to identify hospitalized and non-hospitalized cases of stroke among residents of the city of Dijon, France (156,000 inhabitants).

### 2.2. Stroke Severity and Arterial Occlusion Assessment

Details on clinical signs (consciousness, orientation, gaze, visual disturbance, diplopia, motor or sensory deficit, ataxia, speech disorders, neglect) at first evaluation were registered in medical files by the physician in charge of the patient and systematically reviewed by neurologists from the Dijon Stroke Registry. This first evaluation was performed in the emergency room by emergency doctors and neurologists for almost all patients and by general practitioners for a few outpatients. Stroke severity at onset was quantified using the National Institutes of Health Stroke Scale (NIHSS) score obtained by trained physicians. For each patient, cervical and intracranial arterial imaging exams were performed by neuroradiologists and reviewed by stroke-trained neurologists from the Dijon Stroke Registry to assess the presence and location of the arterial occlusion responsible for the acute IS, as previously described [17].

### 2.3. Stroke Scales to Detect LVO

Sixteen clinical stroke scales to assess clinical severity were considered, where 9 scales were initially designed to detect LVO: the Cincinnati Prehospital Stroke Severity Scale (CP-SSS) [18], the Prehospital Acute Stroke Severity Scale (PASS) [19], the 3-Item Stroke Scale (3ISS) [20], the Stroke vision, aphasia, neglect (VAN) [21], the Large Vessel Occlusion Stroke scale (LVOS) [22], the Gaze-Face-Arm-Speech-Time scale (G-FAST) [23], the Field Assessment Stroke Triage for Emergency Destination (FAST-ED) [24], the Rapid Arterial oCclusion Evaluation (RACE) scale [25], and the Acute Stroke Registry and Analysis of Lausanne (ASTRAL) [26]. Other brief scales not initially designed for the detection of LVO but previously assessed for such a purpose [7] were considered: the Recognition of Stroke in the Emergency Room (ROSIER) scale [27], the mNIHSS [28], the aNIHSS [29], the sNIHSS 5 [30], the NIHSS-R [31], the 3-Item Maria Prehospital Stroke Scale (MPSS) [32], and the shortened NIH Stroke Scale for Emergency Medical Services (sNIHSS-EMS) [33]. The different items of the scales were rated according to the NIHSS score or the first clinical examination for some items not present in the NIHSS score, especially diplopia. The different items used for each scale are reported in Table 1. Individual scores were calculated according to the published risk scoring system for each scale.

### 2.4. Statistical Analyses

For each scale, a receiver operator characteristic (ROC) analysis was performed, and the c-statistic representing the area under the ROC curve was evaluated to assess discrimination for predicting LVO. Cut-off scores presented in a previous review were used to assess sensitivity and specificity and were reported in Table 2 [7]. Because of variations in the definition of LVO between studies, we conducted two series of analyses. In the first series, LVO was defined as an occlusion site affecting the terminal intracranial internal carotid artery (ICA), the M1 segment of the middle cerebral artery (MCA) (including tandem occlusions), or the basilar artery (restricted definition). In the second series, the LVO definition also included the M2 segment of the MCA (wide definition). Statistical analysis was performed with the STATA@13 software (StataCorp LP, College Station, TX, USA).

### 2.5. Ethics

The Dijon Stroke Registry was approved by the following national ethics boards: the Comité d’Evaluation des Registres (French National Committee of Registers), Santé Publique France (French Institute for Public Health Surveillance), and the Commission Nationale Informatique et Libertés (French Data Protection Authority). In accordance with French legislation, the boards waived the need for written patient consent.

## 3. Results

Over the study period, 1060 cases of acute IS were recorded including 971 patients (91.6%) with available data on arterial imaging (median age (IQR): 79 (65–87), 52.9% women, median NIHSS score (IQR): 4 (0–10)). In particular, 836 patients had a computed tomography angiography (CTA); 456 patients had an MRI, among whom 53 had a cervical MRA; 683 patients had a US Doppler of their cervical arteries, among whom 453 had a transcranial Doppler; and 80 patients had a catheter angiography. Among patients with available data on arterial imaging, 284 (29.2%) had a visible arterial occlusion responsible for the acute IS, including 123 (12.7%) patients with LVO according to the restricted definition (Intracranial ICA, MCA until M1 and BA) and 174 (17.9%) patients with LVO according to the wide definition (intracranial ICA, MCA until M2 and BA).

When considering the restricted definition of LVO, the c-statistic for the different scales ranged between 0.66 (aNIHSS) and 0.80 (MPSS) (Table 2).

**Figure 1:**
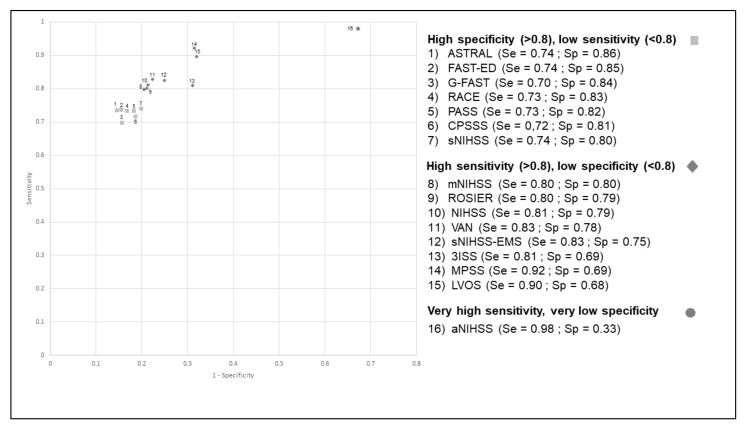
Receiver operating characteristic curve (ROC) for the different scales for the identification of occlusion of the terminal internal carotid artery, the M1 segment of middle cerebral artery, or the basilar artery (wide definition).

**Figure 2:**
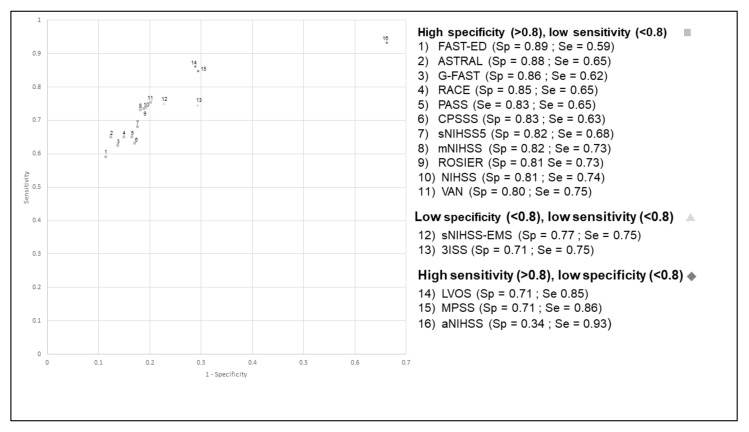
Receiver operating characteristic curve (ROC) for the different scales for the identification of occlusion of the terminal internal carotid artery, the M1 and M2 segments of the middle cerebral artery, or the basilar artery (wide definition).

The aNIHSS scale had the highest sensitivity (98%) but a lower specificity (33%) (Figure 1). Conversely, ASTRAL had the highest specificity (86%) but a low sensitivity (74%). The positive predictive value (PPV) ranged between 17% (aNIHSS) and 41% (ASTRAL), while the negative predictive value (NPV) ranged between 95% (CPSSS, G-FAST) and 99% (aNIHSS). The aNIHSS again had the lowest PPV (17%) but a very high NPV (99%) (Table 3). The c-statistic was slightly lower when considering the wide definition of LVO, ranging between 0.64 and 0.79 (Table 2). The aNIHSS scale had the highest sensitivity (93%) when considering both the restricted and the wide definition of LVO (Figure 1 and Figure 2). Conversely, several scales had a high specificity but a low sensitivity. Their PPV was higher than that observed with the restricted definition, ranging between 23% (aNIHSS) and 52% (FAST-ED and ASTRAL), while the NPV ranged between 91% (CPSSS, FAST-ED) and 96% (aNIHSS, LVOS and MPSS). Our results remained unchanged when analyses were restricted to patients with a time between onset or last proof of good health and the end of clinical and imaging assessment (data not shown).

Detailed information about the clinical examination was available in 116 out of 123 patients with LVO according to the restricted definition. Among these patients, 108 (93%) had at least one cortical sign, including gaze deviation, visual impairment, aphasia, or hemineglect. In 99 patients, cortical signs were accompanied with a motor deficit. When considering the wide definition of LVO, 147 out of 165 patients (89%) with available information had at least one cortical sign, among whom 128 had associated motor impairment.

## 4. Discussion

This study demonstrated that currently available clinical severity stroke scales have very heterogeneous sensitivity and specificity for the detection of LVO, and that none have a good enough combination of both to offer a reliable tool in routine practice.

Compared with previous meta-analyses of studies that assessed the reliability of these brief scales in LVO detection [7,8], we found globally lower AUCs. Such differences may be due to the fact that the scales were designed and previously evaluated in a selected population, mainly patients recruited in stroke units, who are not representative of the whole spectrum of stroke patients. Our population-based design helped to address this issue.

Since the objective of clinical stroke severity scales is to assist clinicians in making decisions regarding the triage of stroke patients, especially in pre-hospital settings, our findings cast doubt on the usefulness of the currently available tools in routine practice. When considering the restricted definition of LVO, the application of the scales that had the highest specificity would miss almost 30% of patients with an actual LVO. Conversely, the use of scales with the highest sensitivity would be associated with an unconfirmed suspicion of LVO in 20% to 30% of patients, and even two-thirds for the aNIHSS scale. The reliability of the scales was even poorer when considering the wide definition of LVO. Because of the limited number of thrombectomy-capable centres in France, as in many other countries, our results seem to indicate that decision making regarding pre-hospital triage could not rely exclusively on these scales since this would expose the system to the risk of excessive workload.

Currently, pre-hospital scales are mostly composed of clinical items including cortical and motor signs. In our population, 11% of patients with LVO (wide definition) had no cortical signs, and 22% had no association between a cortical sign and a motor impairment. Consequently, these patients did not reach the cut-off points of the different scales based on cortical signs and motor deficits. Future scales based on these items alone will have the same flaws, with either a lack of sensitivity or a lack of specificity due to overestimation of the risk of proximal occlusion in the presence of weak symptoms. Another consideration is that, in our study as in others, the assessment of LVO detection scales was performed in patients with a final diagnosis of ischemic stroke. Patients with differential diagnoses including haemorrhagic stroke or stroke mimics were not considered in order to estimate false positive cases, and it could be assumed that the specificity of the scales would be even lower if these patients had been included. According to previous studies, the proportion of stroke mimics was estimated to be between 22% and 42% of suspected stroke patients [34,35,36,37,38]. Notably, it has been suggested that this proportion was similar whether the initial assessment was done by a neurologist or a paramedic [37].

Our study has several strengths. Despite its population-based methodology, imaging coverage was high, with missing data about arterial occlusion in only 8.4% of patients. Detailed information on neurological signs was recorded in the Dijon Stroke Registry, thus making it possible to assess patients’ clinical features accurately. However, some limitations must be acknowledged. In our study, the clinical information was collected at the first clinical examination, i.e., at admission for the majority of the patients. Therefore, it cannot be excluded that some patients had clinical worsening, or improvement, between stroke onset and medical management. Whether patients with LVO are more prone to early changes in their symptoms needs to be assessed. In addition, there may be a gap between the clinical exam performed by a paramedic during the pre-hospital phase and that performed by an emergency doctor or stroke specialist. Such a gap may contribute to a poorer performance of the scales in the pre-hospital phase compared with their performance during an in-hospital evaluation due to difficulties in identifying subtle neurological impairment. However, a recent study showed an excellent reliability in the FAST-ED score obtained by paramedics compared with the score obtained by vascular neurologists [39], thus suggesting that differences in modalities of clinical evaluation may have only a minor impact on our findings. Finally, our study was based on a limited number of patients with LVO, thus limiting its power.

## 5. Conclusions

To conclude, our study demonstrated that currently available clinical stroke severity scales failed to combine both a high sensitivity and specificity to detect LVO in routine clinical practice. These results support the need for further studies to address the issue of the best strategy for pre-hospital triage of IS patients and to assess the effectiveness of alternative management, such as mobile stroke units [40].

## Figures and Tables

**Table 1:** Items used in different scales in order to predict occlusion along with patients with available data in the Dijon Stroke Registry.

Scale	Patients with Data	Gender	AF	mRS	Clinical items													
					Consciousness	Questions	Commands	Gaze	Visual	Diplopia	False Palsy	Arm Motor	Leg Motor	Ataxia	Sensory	Language	Dysarthria	Neglect
CPSSS	949				X			X				x						
ROSIER	949								X		X	X	X			X	X	
RACE	949						X	X			X	X	X					X
ASTRAL	943	X	X	X	X	X	X	X	X		X	X	X	X	X	X	X	X
mNIHSS	938					X	X	X	X			X	X		X	X		X
aNIHSS	949										X	X	X				X	
sNIHSS5	949							X	X				X			X		
PASS	949					X		X				X						
3ISS	949				X			X				X	X					
Van	949						X		X	X		X	X			X		X
LVOS	941							X			X	X	X			X		
MPSS	951										X	X				X	X	
sNIHSS-EMS	941				X						X	X	X		X	X	X	
G-FAST	949							X			X	X				X		
FAST-ED	946							X			X	X				X		X
NIHSS	961				X	X	X	X	X	X	X	X	X	X	X	X	X	X

**Table 2:** Area under the curve (AUC) of the different scales for the restricted and the wide definition of large vessel occlusion (LVO).

Scale	AUC for the Restricted Definition of LVO	AUC for the Wide Definition of LVO
CPSSS	0.765	0.730
ROSIER	0.795	0.772
RACE	0.782	0.749
ASTRAL	0.795	0.761
mNIHSS	0.797	0.774
aNIHSS	0.655	0.635
sNIHSS5	0.772	0.751
PASS	0.775	0.741
3ISS	0.750	0.726
VAN	0.802	0.775
LVOS	0.788	0.777
MPSS	0.804	0.786
G-FAST	0.771	0.743
FAST-ED	0.791	0.737
NIHSS	0.799	0.772
sNIHSS-EMS	0.788	0.761

**Table 3:** Positive predictive value (PPV) and negative predictive value (NPV) of the different scales for the restricted and the wide definition of large vessel occlusion (LVO).

Scale	Restricted Definition of LVO		Wide Definition of LVO	
	PPV	NPV	PPV	NPV
CPSSS	0.35	0.95	0.44	0.91
ROSIER	0.35	0.97	0.45	0.94
RACE	0.38	0.96	0.48	0.92
ASTRAL	0.41	0.96	0.52	0.92
mNIHSS	0.35	0.97	0.46	0.94
aNIHSS	0.17	0.99	0.23	0.96
sNIHSS5	0.34	0.96	0.45	0.92
PASS	0.36	0.96	0.45	0.92
3ISS	0.27	0.96	0.35	0.93
VAN	0.34	0.97	0.44	0.94
LVOS	0.28	0.98	0.38	0.96
MPSS	0.29	0.99	0.39	0.96
G-FAST	0.38	0.95	0.49	0.92
FAST-ED	0.39	0.96	0.52	0.91
NIHSS	0.34	0.97	0.45	0.94
sNIHSS-EMS	0.31	0.97	0.41	0.94

## Data Availability

The authors declare that all supporting data are available within the article.
